# Assessment of Five Pesticides as Endocrine-Disrupting Chemicals: Effects on Estrogen Receptors and Aromatase

**DOI:** 10.3390/ijerph19041959

**Published:** 2022-02-10

**Authors:** Marta Gea, Chao Zhang, Roberta Tota, Gianfranco Gilardi, Giovanna Di Nardo, Tiziana Schilirò

**Affiliations:** 1 Department of Public Health and Pediatrics, University of Torino, 10126 Torino, Italy; roberta.tota@edu.unito.it (R.T.); tiziana.schiliro@unito.it (T.S.); 2 Department of Life Science and Systems Biology, University of Torino, 10123 Torino, Italy; chao.zhang@unito.it (C.Z.); gianfranco.gilardi@unito.it (G.G.); giovanna.dinardo@unito.it (G.D.N.)

**Keywords:** estrogen receptors, aromatase, pesticides, estrogenic activity, gene reporter assay, endocrine-disrupting chemicals, neonicotinoids, estrogen synthesis, estrogen signaling, estrogen equivalency factor

## Abstract

Pesticides are widely applied all over the world, and pesticide exposure can induce different biological effects posing a possible threat to human health. Due to their effects on the endocrine system, some pesticides are classified as endocrine disruptors. The aim of the study is to assess the interference of five pesticides on estrogen biosynthesis and estrogen signaling. Three neonicotinoid insecticides (Acetamiprid, Clothianidin, and Thiamethoxam), a carbamate insecticide (Methiocarb) and a herbicide (Oxadiazon) were tested. The effect of pesticides on estrogen biosynthesis was studied through an ELISA assay using a recombinant form of human aromatase, the enzyme that catalyzes the transformation of androgens to estrogens. Moreover, the effect of pesticides on estrogen signaling was assessed using a gene reporter assay on MELN cells, which measures estrogen receptor-mediated estrogenic activity. The results of the ELISA assay showed that the pesticides did not alter aromatase activity (no interference with estrogen biosynthesis), while the results of the gene reporter assay showed that only Methiocarb was able to alter estrogen signaling at high doses. The estrogenic activity of Methiocarb, expressed as 17β-estradiol equivalency factor (EEF), was equal to 8.0 × 10^−8^. In conclusion, this study suggested that Methiocarb should be considered a potential endocrine disruptor.

## 1. Introduction

During the last century, a significant increase in world food production has become necessary to sustain global population growth [1]. In order to achieve appropriate food quantity and satisfactory food quality, fertilizers and also pesticides have been extensively used.

The term pesticide includes a wide variety of compounds that are used to kill pests, including insects, rodents, fungi and unwanted plants [2]. Pesticides are considered a quick, easy, and inexpensive solution for controlling pests and their use has not only contributed to the leap in agricultural yield, but has also helped to fight vector-borne and food-borne diseases [3,4]. Due to these advantages, great quantities of pesticides are ap-plied every year. In 2019, in the world, more than 4 million tons of pesticides were used [5]. Regarding insecticides, the neonicotinoids are the most widely utilized in the world; in 2014, the neonicotinoid market exceeded USD 3 billion and accounted for about 25% of the global pesticide market [6]. Moreover, pesticides are also extensively applied in agricultural sector; in the European Union alone, the sales of plant protection products per hectare of agricultural area amount to 2.3 kg [3]. The increase in the use of pesticides has been regulated through legislation. In the European Union, previously fragmented legislation was replaced with harmonized pesticide standards for all member states; however, pesticide legislation varies greatly worldwide (more stringent regulations have been approved by developed nations with respect to developing countries) and, to date, harmonized pesticide legislation does not exist [7].

Despite the advantages, the use of pesticides also brings disadvantages. The extensive application of these molecules has ensured their spread in the environment. Numerous studies have demonstrated that air, water and soil can be contaminated by pesticide residues [1,8,9,10,11,12,13]. Once in the environment, pesticides can be accumulated in non-human organisms with devastating toxic effects at population level [14]. Moreover, they can move up trophic chains affecting top predators [1] and the repeated application of pesticides can increase pest resistance. Since the pesticide mechanism for toxic action can be not completely restricted to target pests, toxicity can be exerted also on non-target organisms causing health effects [1]. As a consequence, pesticide toxicity may lead to biodiversity loss and human adverse health effects [2,15]. 

Human exposure to pesticides can occur through the ingestion of foods or liquids containing pesticide residues, through the inhalation of pesticide-contaminated air, or through dermal contact with these molecules [2,16]. Toxic effects induced by pesticide exposure can range from mild symptoms (such as skin irritation) to more severe symptoms (such as headache or nausea). Moreover, some studies reported that pesticide exposure can induce long-term health effects, including cancer [2]. Due to their effects on the endocrine system, some pesticides have also been classified as endocrine-disrupting chemicals (EDCs) [17], namely as molecules that are able to alter the function of the endocrine system causing adverse health effects [18]. The effect of EDCs on the endocrine system is of particular interest since it can be induced by low doses, can be severe when the exposure occurs during childhood or adolescence, can be evident after long time from the time of exposure and can be exerted not only on the exposed individual but also on subsequent generations [18]. At a cellular level, EDCs may interfere with hormone functions in different ways. They can directly interact with hormone receptors, mimicking natural hormones and producing an overstimulation (agonist EDCs) or they can bind hormone receptors, preventing the binding of the endogenous hormone and therefore blocking the signal (antagonist EDCs). Moreover, EDCs may also interfere indirectly with hormones, affecting their synthesis, transport, metabolism and excretion [19].

Chemicals that interfere with estrogens are considered important EDCs. Through direct interaction with receptors, they can alter the estrogenic signaling, which is based on two pathways: genomic pathway and non-genomic pathway. The first pathway involves the transcription of genes and it is initiated by the binding of EDCs with the nuclear estrogen receptors, while the second one is mediated by membrane-bound receptors and it involves signaling proteins [20]. Both pathways can influence different cell functions, such as inflammation response, cellular metabolism, apoptosis, autophagy, DNA damage, and differentiation. Moreover, EDCs that interfere with estrogens can also alter estrogen biosynthesis acting on aromatase enzyme, which is the enzyme that catalyzes the transformation of androgens to estrogens.

Some pesticides have been identified as estrogenic EDCs, such as Dieldrin, which affects cellular proliferation pathway through estrogen receptors and extracellular signal-regulated kinase [21], or Methoxyclor, which affects the apoptosis pathway through the estrogen receptors cyclin D1, Ras, and Bax [22]. Moreover, other pesticides have been tested to assess their activity on aromatase enzyme (e.g., Lindane, Endosulfan, Deltamethrin, Chlorpyriphos, and Atrazine) [23,24,25]. However, for a great number of pesticides, additional evidence is needed. Therefore, the aim of the study is to assess the interference of five pesticides on estrogen biosynthesis and estrogen signaling. Three neonicotinoid insecticides (Acetamiprid, Clothianidin, and Thiamethoxam), a carbamate insecticide (Methiocarb) and a herbicide (Oxadiazon) are tested. The effect of pesticides on estrogen biosynthesis is studied using a recombinant form of human aromatase. Moreover, the effect of pesticides on estrogen signaling is assessed using a gene reporter assay on MELN cells, which measures estrogen receptor-mediated estrogenic activity. 

## 2. Materials and Methods

### 2.1. Pesticides

**Figure 1:**
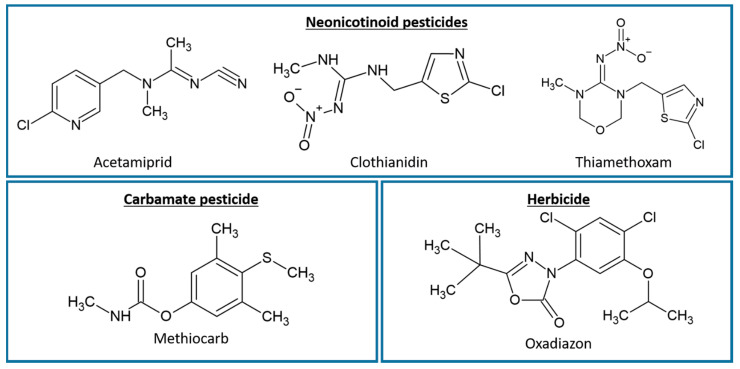
Molecular structures of the five tested pesticides.

Four insecticides and one herbicide were purchased by Merck (Darmstadt, Germany) and tested in order to assess their interference with the aromatase enzyme and estrogen receptors (Figure 1, Table S1 in Supplementary Materials). 

### 2.2. ELISA Assay

The effect of pesticides on estrogen biosynthesis was evaluated using a direct competitive ELISA estrone Kit (BioVendor, Brno, Czech Republic) to quantify estrone produced during an aromatase-catalyzed reaction. The recombinant form of human aromatase and the human recombinant cytochrome P450 reductase (hCPR) were expressed and purified as previously described [26,27]. Reactions were carried out by mixing 5 × 10^−9^ M aromatase, 5 × 10^−9^ M hCPR, 5 × 10^−8^ M androstenedione (Biozol, Eching, Germany), 5 × 10^−4^ M NADPH (VWR International, Milan, Italy), and different concentrations of pesticides (0.5, 1 and 5 × 10^−6^ M) in 100 mM potassium buffer (pH 7.0) containing 20% glycerol (Merck, Darmstadt, Germany), 1 mM β-mercaptoethanol (Merck, Darmstadt, Germany). The reaction was initiated by the addition of NADPH and terminated by heat inactivation at 90 °C for 10 min after incubation at 30 °C for 10 min. The positive control is the reaction without pesticide, and there are two negative controls: the reaction without pesticide but containing anastrozole (aromatase inhibitor), and the reaction without hCPR. After the reaction, the supernatant was obtained by centrifugation at 11,000 g for 10 min, and then diluted with Calibrator A provided by the ELISA kit at 1:8. ELISA was then performed according to the manufacturer’s instructions to measure estrone. The estrone concentration from each reaction was calculated according to a standard curve of known estrone concentrations typically resulting in values ranging from 2.4 to 3.2 nM. All results are expressed as relative aromatase activity with respect to positive control (C+ = reaction without pesticide, relative aromatase activity of C+ = 100%). The relative aromatase activity of each experimental condition was calculated from the average of three measurements, representing at least three independent experiments. 

### 2.3. MELN Gene Reporter Assay

A luciferase gene reporter assay based on the MCF-7 cell lines transfected with the ERE-βGlob-Luc-SVNeo plasmid (MELN cells) was performed in order to assess the estrogenic activity of the pesticides [28,29]. MELN cells, kindly provided by Dr. P. Balaguer (INSERM, Montpellier, France), were grown in 75 cm^2^ flasks at 37 °C and 5% CO_2_ in Dulbecco’s Modified Eagle’s Medium Nutrient Mixture F12-Ham (DMEM-F12) (Merck, Darmstadt, Germany), supplemented with fetal bovine serum (FBS) (Thermo Fisher Scientific, Waltham, MA, USA) (5% *v/v*), penicillin-streptomycin (Biowest, Nuaillé, France) (100 U/mL–100 µg/mL), l-glutamine (Biowest, Nuaillé, France) (4 mM), G418 (Roche Diagnostics, Monza, Italy) (1 mg/mL) and phenol red (Merck, Darmstadt, Germany). Before the assay, the cells were cultured for three days in the test medium, which was DMEM-F12 supplemented with dextran-coated charcoal-treated FBS (Merck, Darmstadt, Germany) (5% *v/v*), penicillin-streptomycin (100 U/mL−100 µg/mL), l-glutamine (4 mM), and without phenol red. Then, the cells were seeded in 96-well plates (40,000 cells/well, 100 µL/well). After 24 h, the cells were exposed to different doses of pesticides for 21 h (Acetamiprid, Clothianidin, and Thiamethoxam from 1.95 × 10^−6^ to 10^−3^ M, Methiocarb and Oxadiazon from 1.95 × 10^−6^ to 10^−4^ M). The pesticide concentrations were selected considering the results of a previous study [29], in which a significant estrogenic effect was induced starting from the concentration 10^−6^ M. Stock solutions of Acetamiprid, Clothianidin, Thiamethoxam, Methiocarb, and Oxadiazon were prepared in dimethyl sulphoxide (DMSO) and stored at −20 °C, while the final dilutions of pesticides were prepared in test medium. The DMSO in the final dilutions was less than 0.1%. At the end of the exposure, the One-Glo Luciferase Assay System reagent (Promega Italy, Milan, Italy) was added in each well, the plates were shaken for 5 min and the luminescence of each well was measured by a luminometer (Infinite Reader M200 Pro, Tecan, Männedorf, Switzerland).

Cells exposed to the test medium and DMSO (<0.1%) were used as a negative control (C−), while seven 17β-estradiol doses (from 10^−12^ to 10^−8^ M) were assessed as standard positive curve of the reference compound (17β-estradiol). Additional tests were carried out on those pesticides that had induced significant estrogenic effect. In particular, these pesticides were also tested together with tamoxifen (estrogen receptor antagonist), to confirm whether the effect was due to estrogen receptor activation, and in combination with 17β-estradiol, to study the interaction between pesticides and 17β-estradiol. DMSO, 17β-estradiol and tamoxifen were purchased by Merck (Darmstadt, Germany).

The estrogenic activity was expressed as relative luciferase activity and it was calculated as a percentage of the activity induced by the treatment with respect to the activity induced by the positive control, 17β-estradiol 10^−8^ M (relative luciferase activity of C− = 0%, relative luciferase activity of 17β-estradiol 10^−8^ M = 100%). All experimental conditions were tested in quadruplicate in two independent experiments (four wells for each independent experiment) and the results were expressed as means and standard deviations. The relative estrogenic potency of each pesticide in comparison with the reference compound (17β-estradiol) was also assessed through the 17β-estradiol equivalency factor (EEF), which was calculated through the formula:

EEF = 17β-estradiol EC50/pesticide EC50(1)

where EC50 = concentrations at which 50% of biological effect is achieved. Data were reported with the relative confidence intervals (IC95%). 

### 2.4. Statistical Analysis

Statistical analysis was performed using IBM SPSS Statistics 25.0 (IBM, Armonk, NY, USA). A probit regression between the relative luciferase activity and log-transformed concentrations of 17β-estradiol or pesticides was applied in order to estimate the EC50. Since data were not normally distributed, the non-parametric Kruskal–Wallis test followed by the post hoc Dunnett’s test was used to assess significant differences vs. negative control. The differences were considered significant with *p*-value < 0.05. 

## 3. Results

### 3.1. Pesticide Effect on Estrogen Biosynthesis

As listed in Table 1, the selected active neonicotinoid insecticides (Acetamiprid, Clothianidin and Thiamethoxam) could not significantly change the enzyme activity at three different concentrations. For the carbamate insecticide Methiocarb, the enzyme activity decreased with the increase in carbamate concentration, but the change was minimal. As a result, all neonicotinoids, Methiocarb and Oxadiazon did not result in significantly altered aromatase activity.

### 3.2. Pesticide Effect on Estrogen Signaling

**Figure 2:**
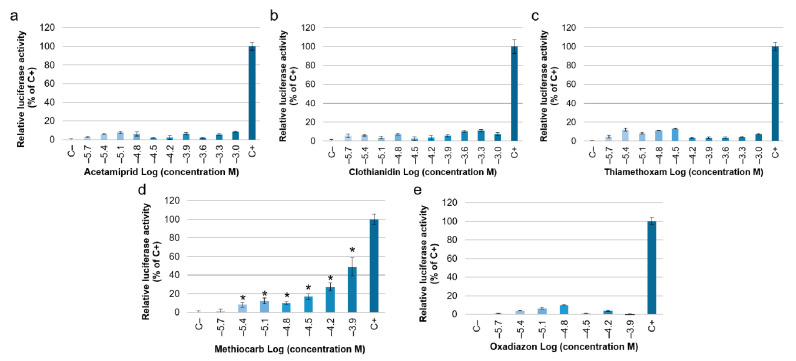
Effect of pesticides on estrogen signaling expressed as estrogenic activity. Estrogenic activity is reported as relative luciferase activity with respect to positive control (C+ = 17β-estradiol 10^−8^ M, relative luciferase activity of C+ = 100%): (**a**) Acetamiprid; (**b**) Clothianidin; (**c**) Thiamethoxam; (**d**) Methiocarb; (**e**) Oxadiazon. Data are presented as means ± standard deviations *: *p*-value < 0.05 vs. negative control (C−) (Kruskal–Wallis followed by post hoc Dunnett’s test).

Pesticide effect on estrogen signaling was assessed using the MELN gene reporter assay. The results showed that the three neonicotinoid insecticides (Acetamiprid, Clothianidin and Thiamethoxam) did not increase the relative luciferase activity with respect to the negative control; therefore, no interference with estrogen signaling was detected (Figure 2a–c). On the contrary, the carbamate insecticide (Methiocarb) induced a dose-dependent estrogenic activity that was significant starting from 3.91 × 10^−6^ M, corresponding to −5.4 Log (concentration M) (Figure 2d). Similar to the neonicotinoid insecticides, the herbicide Oxadiazon did not show any interference with estrogen signaling (Figure 2e).

Since Methiocarb induced a significant estrogenic effect, the concentration 1.25 × 10^−4^ M, corresponding to −3.9 Log (concentration M), was also tested with 17β-estradiol (estrogen receptor agonist) and with tamoxifen (estrogen receptor antagonist). When the pesticide was tested in combination with 17β-estradiol, a higher estrogenic activity than the one induced by 17β-estradiol alone was observed (data not shown). Therefore, in the presence of 17β-estradiol, Methiocarb induced an additive effect on the estrogen receptors. Moreover, when it was tested in combination with tamoxifen, a lower estrogenic activity than the one induced by the pesticide alone was observed, confirming that the observed estrogenic effect was dependent on the estrogen-receptor-mediated pathway. The estrogenic activity of Methiocarb tested together with tamoxifen was higher than the effect induced by the negative control. This result can be explained considering that the pesticide and tamoxifen may compete for estrogen receptor binding. 

The EC50 and the EEF of Methiocarb were 2.1 × 10^−4^ M (IC95% 1.4 × 10^−4^ − 3.8 × 10^−4^ M) and 8.0 × 10^−8^ (IC95% 1.2 × 10^−7^ − 4.5 × 10^−8^), respectively.

## 4. Discussion

Two in vitro assays were applied in this study in order to investigate the interference of four insecticides and one herbicide on estrogen biosynthesis and estrogen signaling. 

All neonicotinoids, Methiocarb and Oxadiazon did not significantly alter aromatase activity; therefore, at the pesticide concentrations used in this study no significant effect on estrogen biosynthesis was found. The results are in accordance with two previous studies [25,30]. The first study showed that Methiocarb did not alter the aromatase activity [25], while the second one underlined that the herbicide Oxadiazon is able to induce aromatase activity in JEG-3 cells [30]. However, the effect of Oxadiazon was found at higher concentrations than the concentrations tested in the present study (effect observed at 10^−5^ M; tested concentrations: 0.5, 1 and 5 × 10^−6^ M). 

Pesticide effect on estrogen signaling was tested using the MELN gene reporter assay; this assay showed that the three neonicotinoid insecticides did not induce any interference on the estrogen signaling (i.e., no estrogenic effect) (tested concentrations: from 1.95 × 10^−6^ to 10^−3^ M). The results obtained by testing Acetamiprid and Thiamethoxam are in accordance with the study of Westlund and Yargeau [31], which assessed the estrogenic activity using a yeast-based in vitro assay (estimated tested concentrations: from 10^−10^ to 10^−2^ M). Moreover, regarding Thiamethoxam, the results are also consistent with the study of Mesnage et al. [32] in which no estrogenic effect was detected testing this pesticide with the E-screen assay on MFC-7 cells (tested concentrations: from 3.4 × 10^−6^ to 1 × 10^−3^ M). Finally, the results obtained testing the last neonicotinoid insecticide, Clothianidin, also agree with a previous study in which the pesticide was tested using the E-screen assay on MFC-7 cells (tested concentrations: from 4 × 10^−6^ to 1.2 × 10^−3^ M) [32]. 

According to data reported by Barbosa et al. [9], environmental concentrations of the three neonicotinoids are lower than the highest concentration tested in this study (Table S2 in Supplementary Materials); therefore, the results showed that the environmental diffusion of the three neonicotinoids does not endanger humans and the environment through the alteration of estrogen signaling. 

In contrast to the neonicotinoids, the insecticide Methiocarb induced a significant estrogenic effect mediated by estrogen receptors (effect observed starting from 3.91 × 10^−6^ M). Similar results were also shown by three previous studies [25,33,34]. Andersen et al. [25] found a significant estrogenic effect testing this pesticide using E-screen assay on MCF-7 BUS cells and a gene reporter assay on transfected MCF-7 BUS cells (effect observed starting from 10^−5^ M and 5 × 10^−6^ M, respectively). In addition, an estrogenic effect was also reported by Kojima et al. [33] and by Tange et al. [34] using gene reporter assays ([33] EC20 = 7.2 × 10^−6^ − 8.4 × 10^−7^ M; [34] EC20 ≈ 2.0 × 10^−5^ M). These studies, together with the results of the present one, confirmed that Methiocarb is able to induce an estrogenic effect in vitro.

The estrogenic effect of Methiocarb was induced only at high doses (starting from 3.91 × 10^−6^ M, corresponding to 880,962.1 ng/L). These doses are higher than the concentrations of this pesticide found in environmental samples (Spain groundwater = 300 ng/L; Mexican groundwater = 5400 ng/L; Spain effluents of wastewaters = 4.73–14.92 ng/L), so its environmental occurrence seems not to represent a threat for human and environment [9,35]. However, it is important to highlight that, in environmental samples, Methiocarb concentrations can be combined with the concentrations of other EDCs causing possible synergistic or additive effects. Therefore, the estrogenic activity of Methiocarb is worthy of attention since it may be combined with the activity of other EDCs at which we are exposed every day.

Finally, in this study, the estrogenic potency of Methiocarb was many orders of magnitude lower than the reference compound and it was lower than the potency of other pesticides (Table 2). However, the EEF of this insecticide was higher than the EEFs of other two neonicotinoids (Imidacloprid and Thiacloprid) that were tested using the same assay in a recent article [29]. 

Regarding the herbicide Oxadiazon, to the best of our knowledge, this is the first article that has assessed the estrogenic activity of this pesticide using an in vitro assay. In these circumstances, it is currently impossible to compare the obtained results with others. However, the result of the present study suggests that this herbicide seems not to induce the transcription of genes regulated by estrogen receptors. This result is in accordance with the review of Ewence et al. [36], in which this pesticide was classified as substance not considered to be an EDC. While no effect on estrogen signaling was found in the present study, a previous study showed that the Oxadiazon-Butachlor pesticide can inhibit the WNT signaling pathway [37]. 

According to data reported by Barbosa et al. [9], environmental concentrations of Oxadiazon are lower than the highest concentration tested in this study (Table S2 in Supplementary Materials); therefore, similar to the three neonicotinoids, Oxadiazon does not endanger humans and the environment through the alteration of estrogen signaling. 

The combined actions of pesticides are very important in the risk assessment process because pesticide formulations may include more than one EDC. Moreover, EDCs can occur in the environment and on the fruits and vegetables we eat as a cocktail of chemicals that may have synergistic, additive, or antagonistic effects on each other. Mixtures of these substances may cause higher toxic effects than those from a single compound [38]. Therefore, more studies are needed to investigate possible additive effects of different pesticides that can be contemporarily found in the environment or on foods as residuals.

As a future perspective, the MELN gene reporter assay and the ELISA assay could be used to evaluate the interference with the estrogen signaling and estrogen synthesis of different pesticide mixtures. Moreover, additional experiments using other in vitro and in vivo assays could be performed in order to confirm the obtained results. For instance, a proliferation assay, such as the E-screen assay, could be performed to evaluate whether these pesticides could induce cell proliferation in estrogen-sensitive cells. 

**Table 2:** Estrogenic potency of Methiocarb assessed in the present study using a gene reporter assay in comparison with estrogenic potency of other pesticides reported by other studies using different assays. Estrogenic potency is expressed as 17β-estradiol equivalency factor (EEF). EEF of 17β-estradiol = 1.

Pesticide	Pesticide Type	Assay	EEF	Reference
Methiocarb	Carbamate insecticide	Gene reporter assay (MELN cells)	8.00 × 10^−8^	Present study
Imidacloprid	Neonicotinoid insecticide	Gene reporter assay (MELN cells)	5.40 × 10^−10^	[29]
Thiacloprid	Neonicotinoid insecticide	Gene reporter assay (MELN cells)	3.70 × 10^−9^	[29]
Chlorpyrifos	Organophosphate insecticide	Yeast Estrogen Screen assay	2.90 × 10^−3^	[39]
Dieldrin	Organochloride insecticide	ER-CALUX assay (T47D Luc cells)	2.40 × 10^−7^	[40]
Endosulfan	Organochloride insecticide	ER-CALUX assay (T47D Luc cells)	1.00 × 10^−6^	[40]
Permethrin	Pyrethroid insecticide	Yeast Estrogen Screen assay	1.00 × 10^−7^—no estrogenic activity	[41]
Chlordane	Organochlorine insecticide	ER-CALUX assay (T47D Luc cells)	9.60 × 10^−7^	[40]
DDT	Organochlorine insecticide	ER-CALUX assay (T47D Luc cells)	9.10 × 10^−6^	[42]
Alachlor	Chloroacetanilide herbicide	Receptor binding assay	8.00 × 10^−6^	[43]

## 5. Conclusions

With world population growth, it has become necessary to increase the production of food. This was partly achieved through the use of various pesticides on a large scale, initially without global guidelines or restrictions. Despite the benefits, these chemicals have polluted almost every part of our environment (i.e., soil, air, and water) and they have entered the trophic chains, reaching top predators. Therefore, the assessment of their environmental persistence and their toxicity for animals as well as humans has become a crucial factor and could be important also for the development of a global pesticide legislation that will protect both humans and the environment. Among the different biological effects that can be induced by pesticides, recently a great deal of attention has been paid to the ability of pesticides to alter the function of the endocrine system.

In this study, the interference of five pesticides (four insecticides and one herbicide) on estrogen biosynthesis and/or signaling, was tested in order to evaluate their potential action as EDCs. As far as we know, this study was the first to assess the effect of Oxadiazon on estrogen receptors using an in vitro assay. The results of the ELISA assay showed that all the four insecticides and the herbicide were not capable of altering aromatase activity; therefore, they did not interfere with estrogen biosynthesis. The results of the gene reporter assay showed that Methiocarb at high doses was able to alter estrogen signaling, while the other tested pesticides showed no estrogenic activity. In conclusion, even if additional in vitro and in vivo studies are needed to confirm this evidence, this study suggests that the carbamate insecticide Methiocarb should be considered as a potential EDC. 

## Figures and Tables

**Table 1:** Effect of pesticides on estrogen biosynthesis expressed as relative aromatase activity with respect to positive control (C+ = reaction without pesticide, relative aromatase activity of C+ = 100%). Data are presented as means ± standard deviations.

Pesticide	Relative Activity (% of C+)		
	0.5 × 10^−6^ M	1 × 10^−6^ M	5 × 10^−6^ M
Acetamiprid	94.7 ± 11.8	99.7 ± 4.7	87.5 ± 17.6
Clothianidin	74.1 ± 24.7	112.9 ± 14.9	97.9 ± 5.2
Thiamethoxam	101.4 ± 13.2	84.1 ± 11.6	95.6 ± 17.1
Methiocarb	98.1 ± 0.2	96.5 ± 0.1	90.9 ± 5.4
Oxadiazon	87.8 ± 0.7	100.1 ± 13.1	101.2 ± 16.7

## Data Availability

The data that supports the findings of this study are available within the article.

## Supplementary Materials

The following are available online at https://www.mdpi.com/article/10.3390/ijerph19041959/s1: Table S1: Characteristics of the five tested pesticides (PubChem database, available at https://pubchem.ncbi.nlm.nih.gov/ (accessed on 2 February 2022)); Table S2: Environmental concentrations of the five tested pesticides in comparison with the concentrations tested in the present study with the MELN gene reporter assay.
