# Supplementary material for: Assessment of Five Pesticides as Endocrine-Disrupting Chemicals: Effects on Estrogen Receptors and Aromatase
Source: Int J Environ Res Public Health. 2022 Feb 10;19(4):1959. doi: 10.3390/ijerph19041959 (PMC8871760; doi:10.3390/ijerph19041959)
Supplement: Supplementary file 1 [file ijerph-19-01959-s001.zip › ijerph-1556872-supplementary.pdf]

SUPPLEMENTARY MATERIALS
Assessment of five pesticides as endocrine-disrupting chemicals: effects on estrogen receptors and aromatase

Marta Gea[1]*, Chao Zhang[2], Roberta Tota[1], Gianfranco Gilardi[2], Giovanna Di Nardo[2] and Tiziana Schilirò[1]

[1] Department of Public Health and Pediatrics, University of Torino, Torino 10126, Italy; roberta.tota@edu.unito.it (R.T.); tiziana.schiliro@unito.it (T.S.).
[2] Department of Life Science and Systems Biology, University of Torino, Torino 10123, Italy; chao.zhang@unito.it (C.Z.); gianfranco.gilardi@unito.it (G.G.); giovanna.dinardo@unito.it (G.D.N.).
* Correspondence: marta.gea@unito.it (M.G.)

Table S.1. Characteristics of the five tested pesticides (PubChem database, available at https://pubchem.ncbi.nlm.nih.gov/).

Name	CAS number	Molecular weight	Molecular formula	Octanol/water partition coefficient (log Kow)	Bioconcentration factor (BCF)
**Acetamiprid**	160430-64-8	222.67	$C_{10}H_{11}ClN_4$	0.8	3
**Clothianidin**	210880-92-5	249.68	$C_6H_8ClN_5O_2S$	0.7	3
**Thiamethoxam**	153719-23-4	291.71	$C_8H_{10}ClN_5O_3S$	-0.13	3
**Methiocarb**	2032-65-7	225.31	$C_{11}H_{15}NO_2S$	2.92	35
**Oxadiazon**	19666-30-9	345.22	$C_{15}H_{18}Cl_2N_2O_3$	4.80	from 24.1 to 708

Table S.2 Environmental concentrations of the five tested pesticides in comparison with the concentrations tested in the present study with the MELN gene reporter assay.

Pesticide	Evironmental concentrations (ng/L) - see references	Tested concentrations using MELN gene reporter assay (ng/L) - present study	Effect on gene reporter assay - present study	References
**Acetamiprid**	Surface water = 20 - 380 ng/L	434,210 - 222,670,000	None	[1]
**Clothianidin**	Surface water = 20 - 420 ng/L	486,880 - 249,680,000	None	[1]
**Thiamethoxam**	Surface water = 40 - 1580 ng/L	568,830 - 291,710,000	None	[1]
**Methiocarb**	Groundwater = 300 - 5,400 ng/L; wastewaters = 4.73 - 14.92 ng/L	439,350 - 22,531,000	effect starting from 880,962.1 ng/L	[1,2]
**Oxadiazon**	Surface water = 4 - 1440 ng/L	673,180 - 34,522,000	None	[1]

References

1. Barbosa, M.O.; Moreira, N.F.F.; Ribeiro, A.R.; Pereira, M.F.R.; Silva, A.M.T. Occurrence and Removal of Organic Micropollutants: An Overview of the Watch List of EU Decision 2015/495. *Water Research* **2016**, *94*, 257–279, doi:10.1016/j.watres.2016.02.047.

2. Jurado, A.; Walther, M.; Díaz-Cruz, M.S. Occurrence, Fate and Environmental Risk Assessment of the Organic Microcontaminants Included in the Watch Lists Set by EU Decisions 2015/495 and 2018/840 in the Groundwater of Spain. *Science of the Total Environment* **2019**, *663*, 285–296, doi:10.1016/j.scitotenv.2019.01.270.
